# Graph theory applied to the analysis of motor activity in patients with schizophrenia and depression

**DOI:** 10.1371/journal.pone.0194791

**Published:** 2018-04-18

**Authors:** Erlend Eindride Fasmer, Ole Bernt Fasmer, Jan Øystein Berle, Ketil J. Oedegaard, Erik R. Hauge

**Affiliations:** 1 Independent researcher, Åndalsnes, Norway; 2 Division of Psychiatry, Haukeland University Hospital, Bergen, Norway; 3 Department of Clinical Medicine, Section for Psychiatry, University of Bergen, Bergen, Norway; 4 K.G. Jebsen Centre for Research on Neuropsychiatric Disorders, Bergen, Norway; Janssen Research and Development, UNITED STATES

## Abstract

Depression and schizophrenia are defined only by their clinical features, and diagnostic separation between them can be difficult. Disturbances in motor activity pattern are central features of both types of disorders. We introduce a new method to analyze time series, called the similarity graph algorithm. Time series of motor activity, obtained from actigraph registrations over 12 days in depressed and schizophrenic patients, were mapped into a graph and we then applied techniques from graph theory to characterize these time series, primarily looking for changes in complexity. The most marked finding was that depressed patients were found to be significantly different from both controls and schizophrenic patients, with evidence of less regularity of the time series, when analyzing the recordings with one hour intervals. These findings support the contention that there are important differences in control systems regulating motor behavior in patients with depression and schizophrenia. The similarity graph algorithm we have described can easily be applied to the study of other types of time series.

## Introduction

Depression and schizophrenia represent major health problems worldwide [1][2]. As is the case with other functional psychiatric disorders, they are defined only by their clinical features, and diagnostic separation between them is not always easy. Depressive symptoms are of course defining features of depressive disorders, but are also common in the course of schizophrenia, and psychotic symptoms are often part of the clinical presentation of depression[3]. It would be an important aid in classification, diagnosis and possibly also in prognostic assessment if one could find objective biological, differences between these disorders. Changes in motor activity are seen both in depression and schizophrenia. Depressed patients differ from control groups regarding gross motor activity, body movements, speech, and motor reaction time [4]. Changes in motor activity are seen both in unipolar depressive disorder[5]and in the depressive phase of bipolar disorder[6]. Motor signs are prominent features in schizophrenia, most clearly seen in catatonia, but psychomotor slowing and extrapyramidal symptoms are also prevalent features [7]. Recording of motor activity with the use of actigraphs is one method that can relatively easily be used in clinical studies. Altered activity patterns have been found both in patients with depression [8] [9]and schizophrenia [7][8]. However, apart from actigraph registrations used in the diagnosis of sleep problems, assessment of motor activity has not been implemented in routine clinical practice.

Since biological systems seldom can be characterized by using simple linear models, we have employed other methods obtained from the fields of non-linear systems, complexity theory and chaos theory to analyze actigraph recordings, and have found differences between patients with depressive disorders and schizophrenia with respect to the organization of motor activity. We have previously reported that by analyzing motor activity over 300 minutes with one minute intervals the schizophrenic patients can be characterized by increased variability in the high frequency compared to the low frequency part of the spectrum using Fourier analysis. At the same time there is an increased complexity of the time series using the sample entropy method [10]. Depressed patients show a different pattern, primarily characterized by increased variability using the standard deviation (SD), and no change in complexity, while manic patients show a pattern that is more similar to the schizophrenic patients [6].

Graph theory has increasingly been applied to the analysis of human brain function [11][12].In this paper we introduce a new heuristic graph algorithm to analyze time series, called the similarity graph algorithm. Time series of motor activity are mapped into a graph, designed to highlight changes in activity such that nodes close to each other that are similar will be connected whereas nodes close to each other that are not similar will not be connected. Then we apply familiar techniques from graph theory to characterize the time series. In recent years several methods have been developed to analyze time series using similar techniques. The first of these methods was the visibility graph (VG) introduced by Lacasa et al. [12]in 2008, and later the horizontal visibility graph [13]. These graphs are designed to discriminate randomness in time series since random time series are mapped to graphs with an exponential degree distribution, independent of the probability distribution from which the series was generated. With these methods important features of time series can be studied, and the methods have been applied to diverse fields, ranging from seismology [14]to the study of human walking rhythm [15]. The similarity graph algorithm presented in this paper is, however, not derived from the visibility graph algorithms, but it is designed independently of them and the gist of the similarity graph algorithm is based on another principle than that of the visibility graph algorithms.

The aims of the present study have been to use this new method to reanalyze our previous actigraph registrations of depressed and schizophrenic patients, both short- and long-term recordings, 1) to see if we can find differences between the two diagnostic groups and the normal controls, 2) to compare this method with the visibility and horizontal visibility graphs, because they may be the most important reference algorithms to compare with when considering the application of graph theory to the study of times series and 3) to look for possible correlations between these findings and altered variability and complexity parameters (sample entropy and rhythm analyses) from our previous studies using the same recordings.

## Materials and methods

### Ethics statement

The study protocol was approved by the Norwegian Regional Medical Research Ethics Committee West. Written informed consent was obtained from all participants involved in the study. The capacity to consent was established by one of the authors (senior psychiatrist).

### Subjects

The study group consisted of 24 psychotic patients (3 women and 21 men), all with a diagnosis of schizophrenia, from an open ward for long-term patients (Knappentunet in Bergen) and 23 patients with mood disorders (10 women and 13 men), all currently depressed, five inpatients from an open psychiatric ward and 18 outpatients, all from the Haukeland University Hospital in Bergen). The control group consisted of 18 women and 11 men, average age 37.8 ± 13.3 years (mean ± SD), range 21–66, medical students (n = 5), patients without serious medical or psychiatric symptoms from a primary care office (n = 4) and employees from Knappentunet (n = 20). None of the control subjects had a history of affective or psychotic symptoms. Patients and controls are reported on in three previous papers [8][10][16].

### Psychiatric assessment

All diagnostic assessments of the depressed patients were performed by one of the authors (OBF) using a semi-structured interview based on DSM-IV criteria [3]for mood disorders.

Diagnostic evaluations of the chronic psychotic patients were made by another of the authors (JØB) and a consensus diagnosis, based on DSM-IV criteria, was made after discussion of each case with OBF.The 23 patients with major depression had mean age 42.8 ±11.0 years. Fifteen had a major depressive disorder, one a bipolar I disorder and 7 bipolar II disorder. None of these patients had psychotic symptoms at the time of the study.The group of 24 patients with schizophrenia had mean age 47.4 ± 11.1 years (range 27–69 years). Their mean age at first hospitalization was 24.4 ± 9.3 years (range 10–52 years). Eighteen had a paranoid form of schizophrenia.Eight of the depressed patients received no psychopharmacological treatment at the time of the study, of the rest (n = 15) all received either one (n = 13) or two (n = 2) antidepressants, five used lithium and one valproate. Five used antipsychotic drugs, mostly in small doses, and three used hypnotics or benzodiazepines. All the schizophrenic patients used antipsychotic drugs, 9 used clozapine, 8 used second generation drugs, 6 traditional antipsychotics, and two a combination of traditional and second generation drugs.

In the depression group affective symptoms were assessed by Montgomery-Asberg Depression Rating Scale (MADRS) scores [17], and in the schizophrenia group symptoms were assessed by the Brief Psychiatric Rating Scale (BPRS) [18].

### Recording of motor activity

Motor activity was monitored with an actigraph worn at the right wrist (Actiwatch, Cambridge Neurotechnology Ltd, England). In the actigraph, activity is measured by using a piezoelectric accelerometer that is programmed to record the integration of intensity, amount and duration of movement in all directions. The sampling frequency is 32 Hz and movements over 0.05 g will be recorded. A corresponding voltage is produced and is stored as an activity count in the memory unit of the actigraph. The number of counts is proportional to the intensity of the movement. The right wrist was chosen to make the procedure more convenient for the participants, since most of them have their watches around the left wrist and it is cumbersome to have two such devices on the same arm. Previous studies have shown that there are only small differences between the right and left wrist [19][20]. Total activity counts were recorded for one minute intervals for a continuous period of at least 12 days for all participants. Patients were instructed to remove the actigraphs when taking a bath or shower, and to record these time intervals. The recorded activity counts (0) in these sequences were then replaced with the mean for the whole recording period.

Since the recordings contain shorter and more prolonged periods of inactivity, each of the time series was searched manually to find periods with continuous motor activity. In particular, recordings from the patients often contained long inactive periods, so in order to use sequences with equal length from all participants we could not use time series longer than 300 min, satisfying the criterion that they contain notmore than 4 consecutive minutes with zero activity.From each participant we then selected one such 300 min period, by searching from the start of the series and using the first period that fulfilled the criteria. Thus we were able to obtain 300 min sequences from each participant. In addition data were analyzed using information from the whole two week period, with activity count for one hour as the unit of measurement.

### Mathematical analyses

#### Graph theory

Graph theory is the science studying graphs, which are mathematical structures that model relations between objects. An undirected graph G = (V, E) consists of a collection V of nodes and a collection E of edges, each of which is a 2-element subset of V that associates two nodes.We thus represent an edge e ∈ E as a two-element subset of V: e = {u, v} for some u, v ∈ V, where we call u and v the ends of e[21]. A directed graph G = (V, E), on the other hand, is different in that every edge e ∈ E has direction and is denoted e = (u, v) for some u, v ∈ V. Note that (u, v) ≠ (v, u).

A graph may exhibit many different topological properties, of which we will only mention the ones relevant to this article. The primary property of a graph lends itself from the very definition a graph. If two nodes *u* and *v* are connected by a single edge, they are said to be adjacent and there exists some kind of symmetric relationship between them if they are part of an undirected graph but an asymmetric relationship if they are part of a directed graph.

The *degree* of a node is the number of nodes it is adjacent to. A *subgraph* of G is a graph formed from a subset of the nodes and edges of G. A *path* in G is a finite sequence of edges connecting a sequence of distinct nodes. Two nodes are said to be *connected* if there exists a path between them. A graph is connected if and only if there is a path between any two nodes in the graph. A connected component H of G is a maximal connected subgraph, that is: H is connected and no nodes from V(G) \ H(G) can be added to H without making a subgraph that is not connected [22]. A directed graph G is said to be *strongly connected* if there is a path following the directions of the edges from every node to every other node. The *strongly connected components* of the graph form a partition into strongly connected subgraphs.

Big O notation is used in computer science to classify algorithms according to the growth rate of their running times or space requirements as functions of the input size. We will only consider the running times of the algorithms described in this paper. Although the notation has a precise mathematical definition, the O notation for a function *f* is usually derived with the following two simplification rules. 1. If *f* is a sum of several terms, only the one with the largest growth rate is kept. 2. If *f* is a product of several factors, any constants that do not depend on the input can be removed. To describe the running time of an algorithm with the big O notation is to give an upper bound on its growth rate. To give some examples, O(1) is the class of algorithms with constant running time, O(log n) is the class of algorithms with logarithmic running time, and O(n) is the class of algorithms with linear running time. Generally, n represents the number of nodes when it comes to graph algorithms, that is, n = |V|.

#### Similarity graph

**Fig 1:**
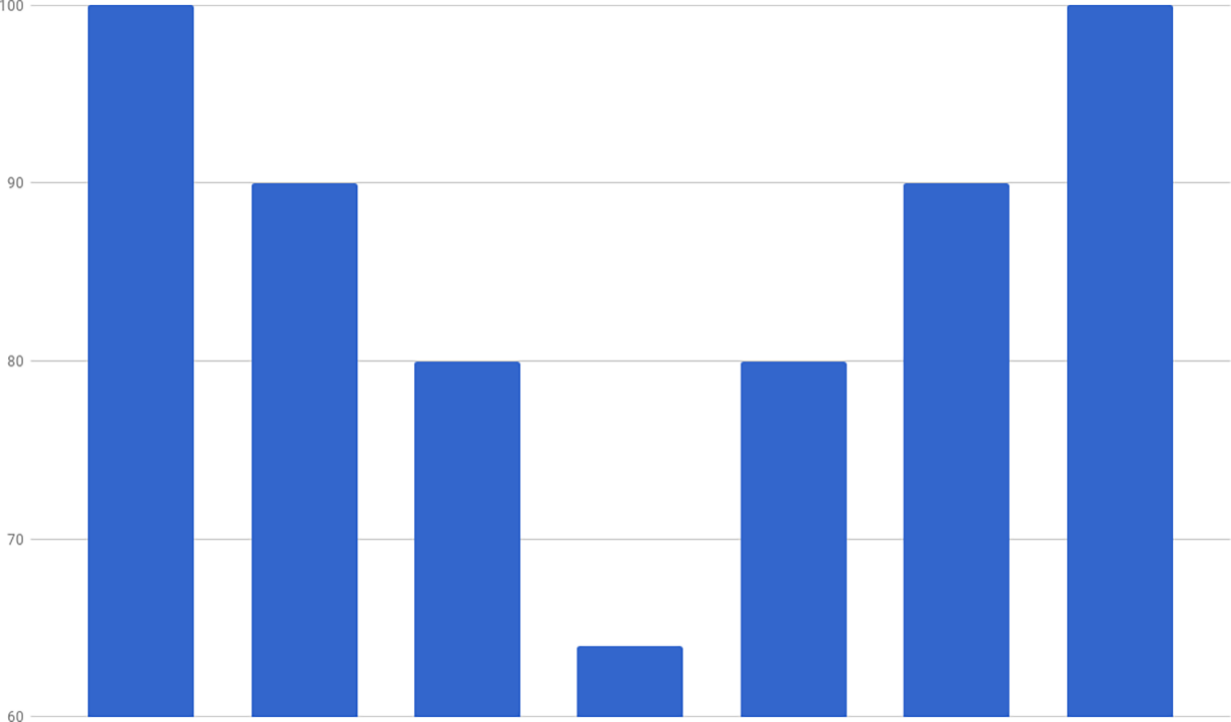
A time series S = (100, 90, 80, 64, 80, 90, 100).

**Fig 2:**
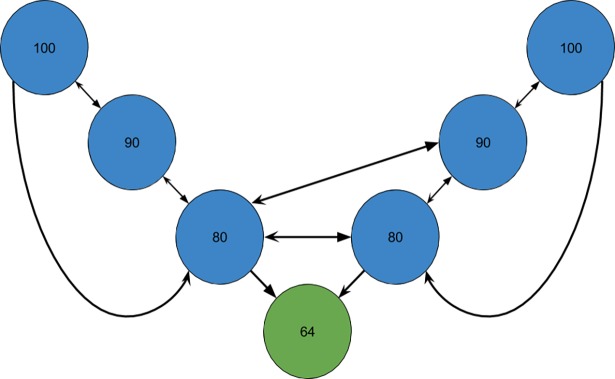
Constructing a directed graph using the asymmetric similarity definition with parameter k = 3 for the time series in Fig 1. The values determining the asymmetric relationships are: 100: [80, 120], 90: [72, 108], 80: [64, 96], 64: [51.2, 76.8]. The graph has two strongly connected components denoted with blue and green colors. Moreover, a directed edge is missing between the 64 node and each of the 80 nodes.

**Fig 3:**
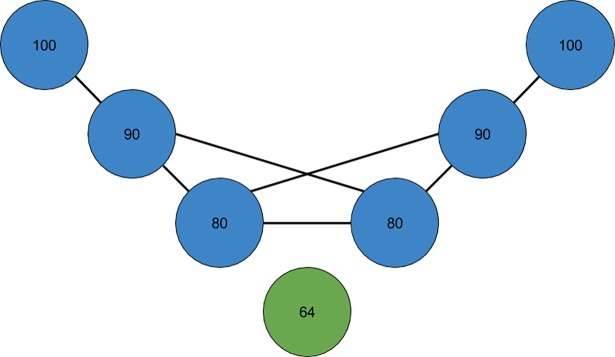
Constructing an undirected graph using the symmetric similarity definition with parameter *k* = 3 for the time series in Fig 1. The values determining the symmetric relationships are: max(100, 90) / min(100, 90) ≈ 1.11 < 1.2. max(100, 80) / min(100, 80) = 1.25 > 1.2. max(90, 80) / min (90, 80) = 1.125 < 1.2. max(80, 64) / min (80, 64) = 1.25 > 1.2. max (90, 64) / min (90, 64) ≈ 1.40 > 1.2. max (100, 64) / min (100,64) ≈ 1.56 > 1.2. The graph has two connected components denoted with blue and green colors. Moreover, the graph misses two edges between directed neighbors.

In this study we apply a new heuristic algorithm that is not examining linear properties of the data, rather, by making a graph, discrete properties of the data series are investigated. Thisalgorithmtransforms a time series S = (x_1_, x_2_, …, x_n_) into a *similarity graph* G = (V,E), either an undirected or a directed graph, depending on the definition of similarity (explained below) used by the algorithm, where each node *u*∈*V =* {1, 2, …, *n*} corresponds to the element x_u_∈ S and where the node *u* is assigned a weight equal to the value of x_u_. The *distance* between two nodes *u* and *v*, is |*u*—*v*|. Two arbitrary nodes *u* and *v* are defined to be *direct neighbors* if their distance is 1. We now introduce two different similarity definitions for nodes, both an asymmetric (a) and a symmetric (b) definition. (a): A node *u* is defined to be *similar* to a node *v* (that is, there is an asymmetric relationship from *u* to *v*)if the weight of *v* is no more than 20% higher or lower than the time interval value of *u*, that is, if x_v_∈ [x_u_—0.2x_u_, x_u_ + 0.2x_u_]. This is a definition that sees the world from *u*’s point of view. (b): Two nodes *u* and *v* are said to be *similar* to each other (that isthey have a symmetric relationship) if max(x_u_, x_v_) / min (x_u_, x_v_) < 1.2. This definition sees the world from the perspective of both *u* and *v*. The directed and undirected similarity graphs are constructed as follows. We introduce a directed edge from one node *u* to another node *v* in the asymmetric case if and only if *u* is similar to *v* and their distance is below a certain threshold *k*. This is illustrated in Figs 1 and 2. We introduce an undirected edge between two nodes in the symmetric case if and only if they are similar to each other and their distance is below a certain threshold *k*. This is illustrated in Figs 1 and 3. The *k* leftmost and the *k* rightmost nodes are disregarded when counting the number of neighbors of each node such that the node of every considered value in *S* may have as much as 2*k* neighbors. Different values of *k* are giving different similarity graphs.

**Fig 4:**
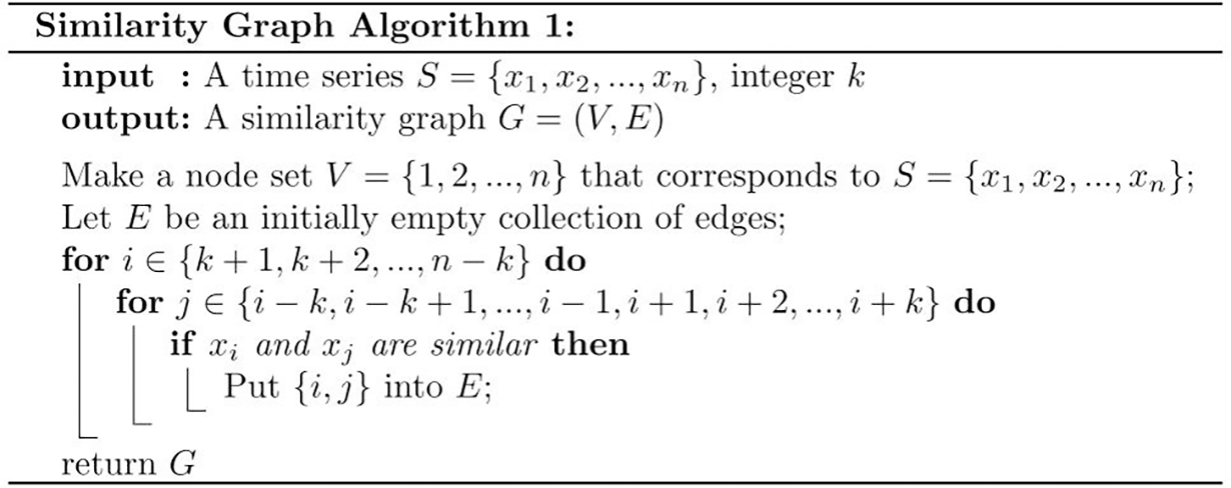
The similarity graph algorithm used in the present study.

The choice of 20% as a threshold for defining two points as similar is based on our previous studies of motor activity with the sample entropy method [10],[23]. The sample entropy method is also based on finding points in time series that are similar to one another, and it is customary to use 20% of the standard deviation for defining two pointsas similar. With the similarity graph method we do not employ the standard deviation, but use 20% of the value of the time points. The standard deviation of these time series from actigraph recordings are usually large, in the order of 100% of the mean, meaning that 20% of the amplitude of an average time point roughly corresponds to 20% of the standard deviation, and we have therefore chosen 20% to define similarity. The rationale for using a threshold *k* as opposed to always considering connecting a node to every other node of the graph is to compare a value in the time series to its nearest past and nearest future in order to obtain a number (i.e. the number of neighbors) designating how much a given value changes compared to its nearest past and future. The higher degree a certain node has, the more similar its weight is to its *k* preceding and *k* subsequent nodes. The lower degree the node has, the more different its value is from its preceding and subsequent values in the corresponding time series. A node with few or no adjacencies indicates a jump in the activity level, either from low to high activity or vice versa. Repeating the algorithm for different values of *k*, gives different similarity graphs, which may reveal different properties of the underlying time series. Another kind of activity jump is revealed by the graph forming connected components when constructing an undirected graph with the symmetric similarity definition and this implies that two periods of time each have smooth changes in activity internally but that the activity changes in one of them are significantly different from the activity changes in the other one. In directed graphs one has to look for strongly connected components, but these have the same properties as the connected components of undirected graphs: They have smooth changes in activity internally. Finally another kind of activity jump is revealed by a pair of direct neighbors in an undirected graph missing a relationship meaning that the activity level in one time interval is significantly smaller or larger than the other. The same property is somewhat more complex in the case with a directed graph since there may be an edge from *u* to *v* but not the other way. This however implies that the weights of *u* and *v* are so far apart that it is only possible to have an edge from the node with the greatest weight to the one with the lowest weight. This also may contribute to the number of strongly connected components in the graph. The formal similarity graph algorithm is given in Fig 4. The running time of the algorithm is O(|V|). We have computed the connected components of the graph with a depth-first search in O(|E|) time [24]. The strongly connected components are computed with Kosaraju’s algorithm in O(|V|+|E|) time.

**Fig 5:**
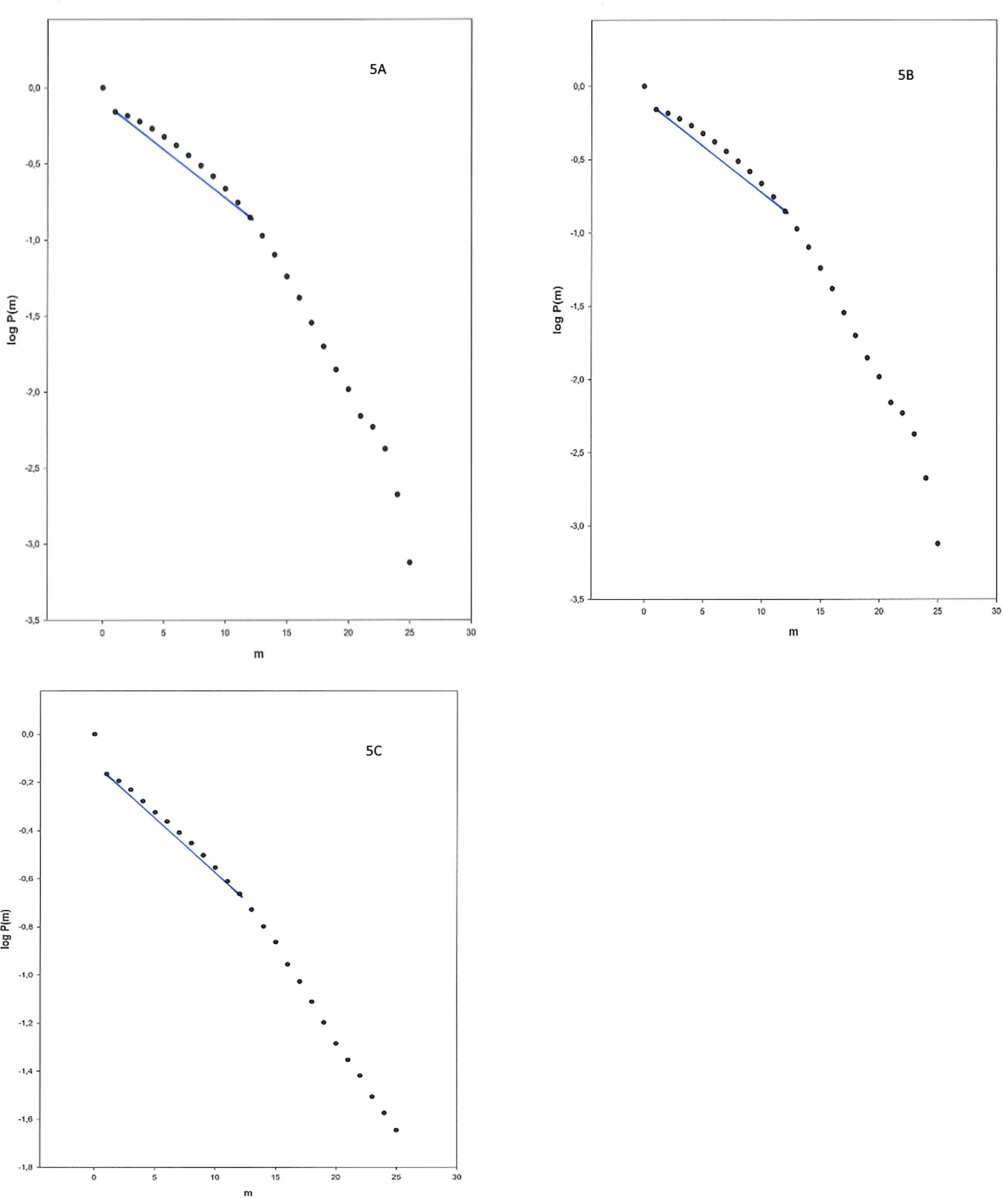
The cumulative probability log P(m) of nodes having m edges (m< 25) plotted on a semilog scale versus m. Controls (A), depressed (B) and schizophrenic (C) patients. The straight line corresponds to values of m(1–12)used for the estimation of the scaling exponent.

With the directed similarity graph we counted the number of edges among the *k* neighbors on each side of the node, using the following values of *k*: 2, 5, 10, 20, 40 and 80. This was done for both the long (12 days) and the short (300 min) time series. We then used the number of neighbors that gave the best separation between the three groups to analyze the same time series with the undirected similarity graph. Following this we calculated the following measures: The mean number of edges, the maximum number of edges, the number of nodes with zero edges (i.e. connected components consisting of a single node), the number of components, the number of missing edges between nearest neighbors, and a scaling exponent, using 40 + 40 neighbors for the 12 days time series and 20 + 20 neighbors for the 300 min time series. Similar to Lacasa et al. [12] we have also looked at the relationship between the number of nodes having m edges and number of edges (m). In each times series there are usually only a few nodes with a high number of edges, while most have an average or below average number. Since we use comparatively short time we have employed the cumulative probability (P) of nodes having m edges (m< 25) and plotted log P(m) versus m on a semilog scale. As can be seen in Fig 5A, 5B and 5C for both controls and patients log P(m) follows a comparatively straight line for values of k from 1 to 12. The scaling exponent is calculated for each person and is defined as the slope of the line that best fit the data (with values of m between 1 and 12) using the least squares method.

The analogy between the similarity graph and symbolic dynamics should be noted [25]. Given a similarity graph on *n* nodes and a fixed number *k*, the maximum number of neighbors of any node is 2*k*, and thus we can map the number of neighbors of each node into a symbol in the alphabet {0,1,2,…,2*k*}. The number *k* must satisfy the inequality 1 ≤ *k* ≤ (*n*-1)/2 when *n* is an odd number and 1 ≤ *k* ≤ (*n*-2)/2 when *n* is an even number. Thus there exists *n*-1 different alphabets when *n* is odd ({0,1,2}, {0,1,2,3,4}, …, {0,1,2,…,*n*-1}) and *n*-2 different alphabets when *n* is even ({0,1,2}, {0,1,2,3,4}, …, {0,1,2,…,*n*-2}). Counting the number of neighbors of a node in the similarity graph with the parameter *k* is the same as calculating the symbol of the time interval in the alphabet {0,1,2,…,2*k*}. However, there is no obvious way of calculating the number of connected components,or other, more complex topological properties of a graph using only symbolic dynamics.

#### Visibility graph

The mean number of edges of the nodes in the similarity graphs constructed in this paper have been compared to corresponding visibility graphs and horizontal visibility graphs of the same time series, since these may be the most important reference graphs to compare with when considering the application of graph theory to the study of time series. The visibility graphs have been constructed using the visibility algorithm described by Lacasa et al. [12]and the horizontal visibility graphs have been constructed using the horizontal visibility algorithm described by Luque et al. [13].

As previously noted, the similarity graph algorithm presented in this paper is designed independently of the visibility graph algorithms and it is based on another principle then them.We will however note the main differences between the similarity graph and the (horizontal) visibility graph.

The main differences between the similarity graph described in this paper and the (horizontal) visibility graph are as follows:The similarity graph algorithm is designed to create a graph that misses edges between pairs of dissimilar nodes and is thus highlighting significant changes in activity (defined by the similarity definition and the 20% margin). The visibility graph algorithm will create an edge between a pair of dissimilar nodes if they can see each other and is thus not suited to highlight changes in activity.The similarity graph may consist of several connected components whereas the (horizontal) visibility graph is always a single connected component since a node in the (horizontal) visibility graph always sees both of its nearest neighbors at a minimum. Thus the number of connected components in the similarity graph will reveal the number of major changes in activity. The (horizontal) visibility graph does not have this ability.The similarity graph has an upper bound on the number of neighbors, whereas a node in the (horizontal) visibility graph may be a neighbor of every other node in the graph. Every pair of direct neighbors in the similarity graph may be missing a relationship (i.e. they are not similar) whereas every pair of direct neighbors in the visibility graph always has a relationship (i.e. they always see each other).Whereas the similarity graph can be constructed in O(|V|) time because each node must be compared to a constant number of other nodes, the visibility graph is constructed in O(|V|^2^) time because the number of edges may be proportional to the square of the number of nodes.

#### Sample entropy

Sample entropy is a nonlinear measure, indicating the degree of regularity (complexity) of time series, and is the negative natural logarithm of an estimate of the conditional probability that subseries of a certain length (m = 2) that match point-wise, within a tolerance (r = 0.2), also match at the next point. Sample entropy is useful for analyses of biological data since it can be employed with comparatively short time series (>50) and is robust with regard to outliers [23].

#### Analyses of rhythms

The variables interdaily stability (IS) and intradaily variability (IV), developed for analysis of actigraphdata, were used [26]. The IS quantifies the invariability between the days, that is, the strength of coupling of the rhythm to supposedly stable environmental factors. The IV indicates the fragmentation of the rhythm, that is, the frequency and extent of transitions between rest and activity.

#### Statistics

One-way Analysis of variance (ANOVA) was employed to evaluate differences between groups, with the p-value set at 0.05, and post hoc Bonferroni tests. Pearson`s correlation coefficient was employed to evaluate correlations, and in addition we have used Analysis of co-variance (ANCOVA) to control for the effect of age and gender. SPSS version 21 was used for the statistical analyses.

## Results

The actigraphicrecordings for 12 days, using one hour sequences, showed that when using the directed similarity graph, and allowing 10, 20, 40 and 80 neighbors in each direction, the mean number of edges from each node was significantly lower for depressed patients compared to controls (Table 1), most pronounced when allowing 40 neighbors (19%lower). For schizophrenic patients there were no significant differences compared to controls. In the 300 min sequences, using the directed similarity graph, there were no significant differences between the three groups for any number of neighbors (Table 2).

**Table 1:** Results from actigraphic recordings for 12 days (288 hrs, 1 hr sequences). Number of edges from each node. Directed similarity graph.

	Control	Depression	Schizophrenia	ANOVA
	(n = 29)	(n = 23)	(n = 24)	
**Number of neighbors**				
4 (2 + 2)	0.755 ± 0.131	0.673 ± 0.141	0.769 ± 0.204	F(73,2) = 2.482, P 0.091
10 (5 + 5)	1.529 ± 0.252	1.347 ± 0.245	1.534 ± 0.425	F(73,2) = 2.734,P = 0.072
20 (10 + 10)	2.295 ± 0.411	1.956 ± 0.353*	2.231 ± 0.595	F(73,2) = 3.733,P = 0.029
40 (20 + 20)	3.685 ± 0.731	3.042 ± 0.632*	3.497 ± 1.026	F(73,2) = 4.160,P = 0.019
80 (40 + 40)	6.436 ± 1.317	5.219 ± 1.069**	6.266 ± 1.766#	F(73,2) = 5.327,P = 0.007
160 (80 + 80)	8.019 ± 1.590	6.692 ± 1.438*	7.891 ± 2.340	F(73,2) = 3.923,P = 0.024

All data are given as mean ± SD. Post hoc Bonferroni tests

* p < 0.05, depression compared to controls

** p < 0.01, depression compared to controls

# p < 0.05, schizophrenia compared to depression

**Table 2:** Results from actigraphic recordings for 12 days (300 min, one min sequences). Number of edges from each node. Directed similarity graph.

	Control	Depression	Schizophrenia	ANOVA
	(n = 29)	(n = 23)	(n = 24)	
**Number of neighbors**				
4 (2 + 2)	0.855 ± 0.206	0.779 ± 0.202	0.865 ± 0.207	F (73,2) = 1.244, P = 0.294
10 (5 + 5)	1.813 ± 0.450	1.634 ± 0.422	1.864 ± 0.41	F (73,2) = 1.788, P = 0.175
20 (10 + 10)	3.103 ± 0.756	2.809 ± 0.701	3.237 ± 0.841	F(73,2) = 1.909, P = 0.156
40 (20 + 20)	5.081 ± 1.250	4.676 ± 1.237	5.501 ± 1.552	F(73,2) = 2.197, P = 0.118
80 (40 + 40)	7.827 ± 2.173	7.405 ± 2.210	8.723 ± 2.892	F(73,2) = 1.819, P = 0.169
160 (80 + 80)	9.219 ± 3.074	8.962 ± 3.363	10.496 ± 3.864	F(73,2) = 1.389, P = 0.256

All data are given as mean ± SD.

We have repeated these analyses with the symmetric version of the program, permitting 40 neighbors in each direction for the 288hr sequences and 20 neighbors for the 300 min sequences. These results are presented in Tables 3 and 4.

**Table 3:** Results from actigraphic recordings for (288 hrs, 1 hr sequences). Number of edges from each node. Undirected similarity graph.

288 hrs				
Number of neighbors				
	Control (n = 29)	Depression (n = 23)	Schizophrenia (n = 24)	ANOVA
80 (40 + 40)	5.785 ± 1.188	4.668 ± 0.989**	5.295 ± 1.284	F(73,2) = 5.898, P = 0.004

All data are given as mean ± SD. Post hoc Bonferroni tests

** p < 0.01, depression compared to controls

**Table 4:** Results from actigraphic recordings for 300 min (one min sequences). Number of edges from each node. Undirected similarity graph.

300 min				
Number of Neighbors				
	Control (n = 29)	Depression (n = 23)	Schizophrenia (n = 24)	ANOVA
40 (20 + 20)	4.478 ± 1.155	3.847 ± 1.295#	4.848 ± 1.497	F(73,2) = 3.494, P = 0.036

All data are given as mean ± SD. Post hoc Bonferroni tests

# p < 0.05, depression compared to schizophrenia

There are shown correlations in Tables 5 and 6, using the directed similarity graph, between measures used in the present study and measures from our previous studies using the same actigraphic recordings, sample entropy and rhythm analyses. In both the long (12 days) and short (300 min) term registrations there are strong negative correlations between the number of edges and the standard deviation and at the same time a positive correlation between sample entropy and the number of edges. For the rhythm analyses (intradaily variability and interdaily stability) there were no significant correlations.

**Table 5:** Actigraphic recordings for 12 days. Correlations between number of edges (40 + 40 neighbors), using the directed similarity graph, and measures of variability and complexity from previous analyses.

		P
Standard deviation (% of mean)	- 0.784	<0.001*
Sample entropy	0.411	<0.001*
Intradaily variability	-0.080	0.498
Interdaily stability	0.108	0.355

*P-value significant using post hoc Bonferroni tests

**Table 6:** Actigraphic recordings for 300 min. Correlations between number of edges (40 + 40 neighbors), using the directed similarity graph, and measures of variability and complexity from previous analyses.

		P
Standard deviation (% of mean)	- 0.725	<0.001*
Sample entropy	0.720	<0.001*

*P-value significant using post hoc Bonferroni tests

When using the visibility graph algorithm and counting the number of edges from each node there were no significant differences between the groups, in either the long term or the short term registrations (Table 7).

**Table 7:** Results from visibility graph analyses. Number of edges from each node.

	Control	Depression	Schizophrenia	ANOVA
	(n = 29)	(n = 23)	(n = 24)	
288 hrs	4.695 ± 0.246	4.754 ± 0.227	4.744 ± 0.329	F(73,2) = 0.364, P = 0.696
300 min	4.257 ± 0.210	4.353 ± 0.223	4.312 ± 0.242	F(73,2) = 1.191, P = 0.310

All data are given as mean ± SD

With the horizontal visibility graph algorithm we found a small, but significant higher number of edges for depressed patients compared to controls (2% higher) for the short term registrations (Table 8).

**Table 8:** Results from horizontal visibility graph analyses. Number of edges from each node.

	Control	Depression	Schizophrenia	ANOVA
	(n = 29)	(n = 23)	(n = 24)	
288 hrs	2.815 ± 0.124	2.797 ± 0.128	2.909 ± 0.639	F(73,2) = 0.626, P = 0.538
300 min	2.831 ± 0.210	2.888 ± 0.094*	2.868 ± 0.072	F(73,2) = 3.970, P = 0.023

All data are given as mean ± SD Post hoc Bonferroni tests

* p < 0.05, depression compared to controls

Fig 5A (controls) shows on a semilog scale the cumulative probability, log P(m), of nodes having m edges (m< 25) vs. m, using the directed similarity graph, 12 days recordings, permitting 40 + 40 neighbors, and with data from all the control persons. Similarly, Fig 5B and 5C show the similar data for depressed and schizophrenic patients.

Table 9 shows additional measures from graph theory for the 12 days recordings, using the directed similarity graph, and permitting 40 + 40 neighbors. For two of the measures, the maximum number of neighbors of each node, andthe scaling exponent, the depressed patients were significantly different from controls, whereas for schizophrenic patients the number of nodes with zero edges was different from controls.

**Table 9:** Results from actigraphic recordings for 12 days (288 hrs, 1 hr sequences), using the directed similarity graph, and 40 + 40 neighbors. Additional measures from graph theory.

	Control	Depression	Schizophrenia	ANOVA
	(n = 29)	(n = 23)	(n = 24)	
Maximum number of edges	22.7 ± 4.4	18.1 ± 3.5	22.5 ± 6.2	F(73,2) = 6.874,P = 0.002
Nodes with zero edges	88.1 ± 3.4	87.9 ± 3.9	91.0 ± 4.3*	F(73,2) = 4.777,P = 0.011
Scaling exponent	-0.046 ± -0.	0.070 ± -0.029**	-0.049 ± -0.029	F(73,2) = 6.398,P = 0.003

All data are given as mean ± SD. Post hoc Bonferroni tests

* p < 0.05, schizophrenia compared to depression or controls

** p < 0.01, depression compared to controls and to schizophrenia

For the short term recordings (300 min), using the directed similarity graph, and 20 + 20 neighbors, there were no significant differences for any of these additional measures (Table 10).

**Table 10:** Results from actigraphic recordings for 300 min (one min sequences), using the directed similarity graph, and 20 + 20 neighbors. Additional measures from graph theory.

	Control	Depression	Schizophrenia	ANOVA
	(n = 29)	(n = 23)	(n = 24)	
Maximum number of edges	27.1 ± 7.3	24.7 ± 6.5	27.8 ± 7.4	F(73,2) = 1.166, P = 0.317
Nodes with zero edges	85.6 ± 1.7	86.2 ± 3.0	85.3 ± 2.7	F(73,2) = 0.699, P = 0.500
Scaling exponent	-0.050 ± -0.024	-0.056 ± -0.032	-0.039 ± -0.023	F(73,2) = 2.454, P = 0.093

All data are given as mean ± SD.

Table 11 shows the number of connected components and missing edges between direct neighbors from actigraphic recordings for 12 days (40 + 40 neighbors), using the undirected similarity graph. The number of components were significantly higher in the schizophrenic patients, but there were no differences for missing edges between direct neighbors.

**Table 11:** Missing edges between direct neighbors from actigraphic recordings for 12 days (288 hrs, 1 hr sequences), with both the directed and the undirected similarity graph, using 80 (40 + 40) neighbors. For the undirected similarity graph number of components are also given.

		Control		Depression	Schizophrenia		ANOVA
		(n = 29)		(n = 23)	(n = 24)		
**Directed**							
Missing edges		243.5 ± 9.5		248.6 ± 10.1	242.5 ± 11.9		F(73,2) = 2.291, P = 0.108
**Undirected**							
Components		8.5 ± 6.6		79.5 ± 12.2	89.8 ± 20.0*		F (73,2) = 5.161, P = 0.008
Missing edges		247.6 ± 8.6		253.2 ± 10.1	250.1 ± 7.8		F (73,2) = 2.646, P = 0.078

All data are given as mean ± SD. Post hoc Bonferroni tests

* p < 0.05, schizophrenia compared to depression or controls

Table 12 shows the number of connected components and missing edges between direct neighbors from actigraphic recordings for 300 min (20 + 20) neighbors, using the undirected similarity graph. The number of connected components were significantly higher in the depressed patients. There were no significant differences for missing edges between direct neighbors.

**Table 12:** Missing edges between direct neighbors from actigraphic recordings for 300 min (one min sequences), with both the directed and the undirected similarity graph, using 40 (20 + 20) neighbors. For the undirected similarity graph number of components are also given.

	Control	Depression	Schizophrenia	ANOVA
	(n = 29)	(n = 23)	(n = 24)	
**Directed**				
Missing edges	238.0 ± 16.3	252.7 ± 15.9	234.3 ± 15.2	F(73,2) = 1.349, P = 0.266
**Undirected**				
Components	73.2 ± 15.2	90.8 ± 23.0**	71.7 ± 19.5	F(73,2) = 7.319, P = 0.001
Missing edges	245.6 ± 15.0	252.7 ± 15.9	242.0 ± 14.9	F(73,2) = 3.004, P = 0.056

All data are given as mean ± SD. Post hoc Bonferroni tests

** p< 0.01, depression compared to controls and to schizophrenia

# p < 0.05, depression compared to schizophrenia

When comparing males and females, using Pearsons correlation coefficient, there were no significant correlations for any of the parameters obtained from use of the similarity graph, when analyzed across the three groups, but the horizontal visibility graph had a weak correlation (0.267, p = 0.020) for the short term recordings (S1 Table). There were no significant correlations for any of the parameters obtained from use of the similarity graph with age, but the visibility graph had a weak correlation (-0.277, p = 0.015) for the long term recordings (S2 Table).Using ANCOVA to control for the effects of age and gender, there were no effects for either age or gender on any of the measures.

Among the patients with schizophrenia we compared those scoring above 56 (median value) on the BPRS to those scoring below 56 with regard to the number of nodes with zero edges (Table 9) and the number of components (Table 11) in the 12 days recordings, and there were no differences between these groups. We also compared the 9 schizophrenic patients that used clozapine to the 15 that used other antipsychotics, and again there were no difference between these groups.

When comparing depressed patients with a bipolar diagnosis to those with unipolar depression there were no significant difference for any of the measures, nor were there any difference on any measure between depressed patients scoring above 24 (median value) on the MADRS compared to those scoring below 24.

## Discussion

The main finding of the present study is that when analyzing motor activity using actigraph registrations, depressed and schizophrenic patients were distinctly different from control persons. We used a new method to evaluate time series, based on techniques from graph theory, constructing directed and undirected similarity graphs using asymmetric and symmetric similarity definitions respectively. There were also significant differences between the two patient groups. This supports the contention that there are important differences in control systems regulating motor behavior in patients with depression and schizophrenia [10].

In the depressed patients each point in these time series, giving the total activity count over one hour and represented by a node in the similarity graph, is connected to fewer neighbors in the similarity graph. It therefore seems that in depressed patients the organization of these time series is characterized by less regularity. The difference from controls was most marked when permitting 40 neighbors before and 40 after the index node. We have no obvious explanation of why this number of neighbors gives the clearest separation of the groups. In addition two other measures were clearly different in the depressed patients, the maximum number of edges was lower, and a scaling exponent (logP(m) vs. m) was more negative, which is a consequence of fewer nodes with a high number of edges. The results for both of these measures thus reflect time series with less regularity.

When constructing the undirected similarity graphs using the symmetric similarity definition we found the same pattern of results when counting the number of neighbors of each node. However, in the 12 days recordings this algorithm produced a more pronounced difference between the groups, and in addition there was a significant difference between groups also for the 300 min sequences, again with lower values for the depressed patients, reflecting time series with less regularity.

With the 12 days recordings the schizophrenic patients had a higher value for nodes with zero edges compared to both controls and depressed patients. However, the difference was small (3% higher) and therefore probably of little importance.

We investigated two additional measures from graph theory, the number of connected components in the undirected graphs using the symmetric similarity definition, and the number of missing edges between direct neighbors. For missing edges between direct neighbors there were no differences between the groups with either the directed or undirected similarity graph in the 12 days and 300 min sequences. A difference between the groups was detected in the number of connected components in the undirected similarity graphin the 12 days recordings, with a substantially higher number of components in the schizophrenic patients. In contrast, for the 300 min recordings the undirected similarity graph showed that the depressed patients had a significantly higher number of components. Such an increased number of components indicates that the patients more often rapidly change their behavior either to a lower or a higher level than before. However, between such changes the behavior only changes smoothly. We have no clear explanation of why the schizophrenic patients differ from the other groups in the long time series while the depressed patients are different in the short term registrations, but we have seen similar differences between these groups when we have used Fourier analysis and other measures of variability [10]. Even though the level of motor activity and activity patterns are changed in both these disorders the mechanisms that are involved may be very different. In depressed patients psychomotor retardation is often a prominent clinical feature, related to functional deficits in the prefrontal cortex and abnormalities in dopamine neurotransmission [27]and may reflect changes in arousal [28]. In schizophrenia symptoms related to motor control are more varied, including neurological soft signs, abnormal involuntary movements and catatonia, and involve several distinct brain networks [29]. In this study we are not able to relate the changes we have found in motor activity to anatomical localisations or physiological parameters in the brain.

In a previous paper on mathematical analyses of motor activity time series, we reported a strong negative correlation between sample entropy values and SD [10], but we were not able to discriminate between patients with depression or schizophrenia and controls, neither in the 300 min or the 288 hrs time series using the sample entropy method. In the present study the mean number of edges also showed a strong negative correlation to the SD, both in the short and the long time series. However there is a strong positive correlation between the number of edges and the sample entropy value, meaning that a high number of edges (low complexity) was associated with a low probability of finding matching patterns in the sample entropy test (high complexity). This finding is intriguing and suggests that the concept of complexity in these time series is not straightforward, and clearly depend on the method used. This is also illustrated by the findings obtained from using von Somerens rhythm analysis with the same data set [8]. In this method one hour sequences are also the basis for the calculations, and we found no clear indications of altered complexity in the depressed patients, but the schizophrenic patients had a more regular pattern (reduced complexity) compared to controls. The two measures, intradaily variability and interdaily stability, did not show any significant correlations with the number of edges calculated in the present study, so these measures apparently capture different aspects of the complexity and variability of time series.

The similarity graph algorithm was designed to transform time series to graphs that may uncover important properties of the time series regarding changes in activity. The visibility [12] and horizontal visibility graph [13] algorithms are probably the most well known algorithms that transform time series to graphs, however in a quite different way than the similarity graph algorithm.The visibility graph has been useful in several other settings [14][15], including analyses of one aspect of motor activity, the human gait rhythm [15]. However, we did not find any differences between ourclinical groups and controls with this method. With the horizontal visibility graph algorithmthe depressed patients showed a small increase in the number of edges in the 300 min recordings compared to controls, but no difference in the 288 hrs recordings. However, this increase in the number of edges with the 300 min recordings was very small (2%) and probably of no clinical relevance, and the lack of effect in the 288 hrs recording are noteworthy compared to the reduction in the number of edges (19%) we found in the 288 hrs recordings with our undirected similarity graph method.

It is therefore clear that in relation to actigraph recordings our similarity graph algorithm reveals differences between clinical groups that are not apparent when using either the visibility graph or horizontal visibility graph algorithms.

It has been proposed that the horizontal visibility graph can be used to distinguish time series with deterministic (chaotic) patterns from stochastic dynamics [30]. We have not tried to use the present similarity graph algorithm in relation to artificial time series with known properties (deterministic or stochastic), and we are therefore not able to say if the time series from our depressed patients are different from controls regarding possible underlying deterministic structures. Short and noisy time series in biology and medicine are inherently difficult to study, but in one previous paper of bipolar patients analyses of mood recordings over extended time periods (2 years) showed evidence of underlying low-dimensional chaotic dynamics by estimation of correlation dimension [31]. However, interpretation of such findings are difficult [32], and we have not tried to use correlation dimension on the present dataset.

It is possible to envisage that by combining different mathematical methods, analyses of actigraph registrations may give a biological “signature” that can be useful for diagnostic purposes. In addition to the present similarity graph algorithmwe would suggest using SD, RMSSD, Fourier analysis, analysis of rhythms and distribution of active and inactive periods [16]. It would obviously be important if clinical impressions and rating scale thus could be supplanted with objective registrations of motor behavior. One further possibility is that such measures can be used to predict treatment effects.

There are several limitations to the present study. Foremost is of course the possibility thattreatment with psychotropic medication may have influenced the results. It is however difficult to separate such an influence from real biological differences between the groups. The depressed patients used a range of different medications, making comparisons difficult, but in the schizophrenia group it was possible to look at differences between those patients using clozapine and those that used other antipsychotics. This comparison revealed no significant differences, in contrast to a previous study with the same patient group, where the clozapine treated patients were clearly different, with regard to analyses of rhythms [8].

The gender distribution is different in the three groups and this could be a possible confounding factor. However, using the similarity graph, we did not find differences between genders for any of the measures we analyzed.

The patients with depression were not very ill, as shown by the MADRS scores, and there was a comparatively small range of scores. It is of course possible that we could have found differences between subgroups if we had included more severely depressed inpatients in our sample. On the other hand, the schizophrenia group was comprised of chronic patients, and it would have been desirable also to have patients with a shorter course of illness to compare with.We did not find any significant differences on any measure between patients with bipolar disorder (mostly bipolar II) and unipolar depressed patients, but the groups are small, and we would clearly need a larger sample to decide on this issue.

The mean age of the schizophrenic patients is higher than the other two groups, and this may also be a relevant confounding factor. However, again we did not find any significant correlations between age and any of the measures.

We have not analyzed sleep parameters, and sleep may be altered both in depressed [33][34] and schizophrenic patients [34]. This may have influenced the findings, but it would be very difficult to separate these effects from other effects on rest and activity rhythms. The controls in our study were employed and working, or students, while the patients were not. This is a source of bias that is difficult to evaluate. Participants wereasked to remove the actigraphs while taking a bath or showering, but this comprises only short time periods, and we think that this is unlikely to have biased the results. A more general constraint is that we have only been able to record total activity count, and therefore do not have any data on velocity or direction of movement.

### Conclusion

In this paper, we have presented a new algorithm to analyze time series of motor activity based on graph theory, which we call the similarity graph algorithm. The approach is based on graph theory and algorithms not hitherto used to analyze motor activity.

The concept of similarity between nodes is crucial in the algorithm, and we have used two different versions of similarity, an asymmetric and a symmetric definition, corresponding to directed and undirected graphs respectively. In these graphs we have investigated well known graph properties and interpreted them with respect to what the graphs are modelling. The two kinds of graphs gave similar results, but the results were slightly more significant when using the symmetric similarity definition.

We found differences between depressed and schizophrenic patients and between these patient groups and controls, but the most marked finding was that depressed patients showed evidence of increased complexity of the time series. This similarity graph algorithm can easily be applied to the study of other types of data, and can be used both to find differences between patient groups and to explore the underlying structure of time series.

## Supporting information

S1 Table The relations between gender and the different parameters reported on in the paper using Pearson correlations. (DOCX)

S2 Table The relations between age and the different parameters reported on in the paper using Pearson correlations. (DOCX)

S1 File Background data for the results from the long term recordings. (XLSX)

S2 File Background data for the results from the short term recordings. (XLSX)
