# Supplementary material for: Graph theory applied to the analysis of motor activity in patients with schizophrenia and depression
Source: PLoS One. 2018 Apr 18;13(4):e0194791. doi: 10.1371/journal.pone.0194791 (PMC5905887; doi:10.1371/journal.pone.0194791)
Supplement: S1 Table — (DOCX) [file pone.0194791.s001.docx]

**S1 Table. The relations between gender and the different parameters reported on in the paper using Pearson correlations.**

**A**

| Results from actigraphic recordings for 12 days (288 hrs, 1 hr sequences). Number of edges from each node. Directed similarity graph. PC = Pearson correlations, P = p-value. |
| --- |
|  |
| **Number of neighbors PC P** |
| 4 (2 + 2) 0.077 0.508 |
| 10 (5 + 5) 0.081 0.488 |
| 20 (10 + 10) 0.073 0.531 |
| 40 (20 + 20) 0.074 0.524 |
| 80 (40 + 40) 0.085 0.464 |
| 60 (80 + 80) 0.129 0.266  **B**  Results from actigraphic recordings for 12 days (300 min, one min sequences). Number of edges from each node. Directed similarity graph. PC = Pearson correlations, P = p-value.   \| **Number of neighbors PC P** \| \| --- \| \| 4 (2 + 2) -0.067 0.566 \| \| 10 (5 + 5) -0.043 0.714 \| \| 20 (10 + 10) -0.016 0.893 \| \| 40 (20 + 20) 0.014 0.907 \| \| 80 (40 + 40) -0.001 0.991 \| \| 160 (80 + 80) 0.004 0.973  **C**  Results from actigraphic recordings for (288 hrs, 1 hr sequences). Number of edges from each node. Undirected similarity graph. PC = Pearson correlations, P = p-value.   \|  \| \| --- \| \| **Number of neighbors PC P** \| \| 80 (40 + 40) 0.026 0.822  **D** \| \|  \| \| Results from actigraphic recordings for 300 min (one min sequences). Number of edges from each node. Undirected similarity graph. PC = Pearson correlations, P = p-value.  **Number of neighbors PC P**  40 (20 + 20) 0.021 0.860 \|  \| **E**  Results from visibility graph analyses. Number of edges from each node. PC = Pearson correlations, P = p-value.  **PC P**   \| 288 hrs 0.027 0.815 \| \| --- \| \| 300 min 0.099 0.394  **F**  Results from horizontal visibility graph analyses. Number of edges from each node. PC = Pearson correlations, P = p-value.  **PC P**   \| 288 hrs 0.103 0.378 \| \| --- \| \| 300 min 0.267 0.020 \|   **G**  Results from actigraphic recordings for 12 days (288 hrs, 1 hr sequences), using the directed similarity graph, and 40 + 40 neighbors. Additional measures from graph theory. PC = Pearson correlations, P = p-value.  **PC P**   \| Maximum number of edges -0.006 0.956 \| \| --- \| \| Nodes with zero edges 0.115 0.321 \| \| Scaling exponent 0.008 0.946 \|  \| **H**  Results from actigraphic recordings for 300 min (one min sequences), using the directed similarity graph, and 20 + 20 neighbors. Additional measures from graph theory. PC = Pearson correlations, P = p-value.  **PC P**   \| Maximum number of edges -0.065 0.580 \| \| --- \| \| Nodes with zero edges 0.094 0.418 \| \| Scaling exponent -0.029 0.804  **I**  Missing edges between direct neighbors from actigraphic recordings for 12 days (288 hrs, 1 hr sequences), with both the directed and the undirected similarity graph, using 80 (40 + 40) neighbors. For the undirected similarity graph number of components are also given. PC = Pearson correlations, P = p-value.  **PC P**   \| **Directed** \| \| --- \| \| Missing edges -0.063 0.587 \| \| **Undirected** \| \| Components 0.076 0.513 \| \| Missing edges 0.013 0.909  **J**  Missing edges between direct neighbors from actigraphic recordings for 300 min (one min sequences), with both the directed and the undirected similarity graph, using 40 (20 + 20) neighbors. For the undirected similarity graph number of components are also given. PC = Pearson correlations, P = p-value.   \| **PC P**   \| **Directed** \| \| --- \| \| Missing edges 0.069 0.554 \| \| **Undirected** \| \| Components 0.019 0.870 \| \| Missing edges 0.038 0.745 \| \| \| --- \| --- \| --- \| --- \| --- \| --- \| \|  \| \|  \| \|  \| \|  \| \|  \| \|  \| \|  \|  \| \| --- \| \|  \| \|  \|  \| \| --- \| \|  \| \|  \| \|  \| \|  \|  \|  \| \| --- \| \|  \| \|  \| \|  \| \|  \| \| \| --- \| --- \| --- \| --- \| --- \| --- \| --- \| --- \| --- \| --- \| --- \| --- \| --- \| --- \| --- \| --- \| --- \| --- \| --- \| --- \| --- \| --- \| --- \| --- \| --- \| --- \| --- \| --- \| --- \| --- \| --- \| --- \| --- \| \|  \| \|  \| \|  \| \| \| \| --- \| --- \| --- \| --- \| --- \| --- \| --- \| --- \| --- \| --- \| --- \| --- \| --- \| --- \| --- \| --- \| --- \| --- \| --- \| --- \| --- \| --- \| --- \| --- \| --- \| --- \| --- \| --- \| --- \| --- \| --- \| --- \| --- \| --- \| --- \| --- \| --- \| --- \| --- \| --- \| --- \| --- \| --- \| --- \| \| \|  \| \| --- \| \|  \| \|  \| \|  \| \| \|  \|  \| \| --- \| \|  \| \|  \| \|  \| \|  \| \|  \| \|  \| \|  \| \|  \| |
