# Supplementary material for: Graph theory applied to the analysis of motor activity in patients with schizophrenia and depression
Source: PLoS One. 2018 Apr 18;13(4):e0194791. doi: 10.1371/journal.pone.0194791 (PMC5905887; doi:10.1371/journal.pone.0194791)
Supplement: S2 Table — (DOCX) [file pone.0194791.s002.docx]

**S2 Table. The relations between age and the different parameters reported on in the paper using Pearson correlations.**

**A**

| Results from actigraphic recordings for 12 days (288 hrs, 1 hr sequences). Number of edges from each node. Directed similarity graph. PC = Pearson correlations, P = p-value. |
| --- |
|  |
| **Number of neighbors PC P** |
| 4 (2 + 2) 0.070 0.550 |
| 10 (5 + 5) 0.072 0.539 |
| 20 (10 + 10) 0.072 0.534 |
| 40 (20 + 20) 0.128 0.270 |
| 80 (40 + 40) 0.156 0.178 |
| 160 (80 + 80) 0.177 0.127  **B**  Results from actigraphic recordings for 12 days (300 min, one min sequences). Number of edges from each node. Directed similarity graph. PC = Pearson correlations, P = p-value.   \| **Number of neighbors PC P** \| \| --- \| \| 4 (2 + 2) -0.067 0.564 \| \| 10 (5 + 5) -0.004 0.970 \| \| 20 (10 + 10) 0.022 0.850 \| \| 40 (20 + 20) -0.004 0.970 \| \| 80 (40 + 40) -0.044 0.707 \| \| 160 (80 + 80) -0.067 0.566  **C**  Results from actigraphic recordings for (288 hrs, 1 hr sequences). Number of edges from each node. Undirected similarity graph. PC = Pearson correlations, P = p-value.   \|  \| \| --- \| \| **Number of neighbors PC P** \| \| 80 (40 + 40) 0.028 0.809  **D** \| \|  \| \| Results from actigraphic recordings for 300 min (one min sequences). Number of edges from each node. Undirected similarity graph. PC = Pearson correlations, P = p-value.  **Number of neighbors PC P**  40 (20 + 20) 0.023 0.847 \|  \| **E**  Results from visibility graph analyses. Number of edges from each node. PC = Pearson correlations, P = p-value.  **PC P**   \| 288 hrs -0.277 0.015 \| \| --- \| \| 300 min -0.069 0.555  **F**  Results from horizontal visibility graph analyses. Number of edges from each node. PC = Pearson correlations, P = p-value.  **PC P**   \| 288 hrs -0.095 0.414 \| \| --- \| \| 300 min -0.036 0.755 \|   **G**  Results from actigraphic recordings for 12 days (288 hrs, 1 hr sequences), using the directed similarity graph, and 40 + 40 neighbors. Additional measures from graph theory. PC = Pearson correlations, P = p-value.  **PC P**   \| Maximum number of edges 0.153 0.188 \| \| --- \| \| Nodes with zero edges 0.082 0.479 \| \| Scaling exponent 0.168 0.146 \|  \| **H**  Results from actigraphic recordings for 300 min (one min sequences), using the directed similarity graph, and 20 + 20 neighbors. Additional measures from graph theory. PC = Pearson correlations, P = p-value.  **PC P**   \| Maximum number of edges -0.114 0.326 \| \| --- \| \| Nodes with zero edges -0.042 0.720 \| \| Scaling exponent 0.117 0.314  **I**  Missing edges between direct neighbors from actigraphic recordings for 12 days (288 hrs, 1 hr sequences), with both the directed and the undirected similarity graph, using 80 (40 + 40) neighbors. For the undirected similarity graph number of components are also given. PC = Pearson correlations, P = p-value.  **PC P**   \| **Directed** \| \| --- \| \| Missing edges 0.020 0.865 \| \| **Undirected** \| \| Components 0.099 0.394 \| \| Missing edges 0.177 0.127  **J**  Missing edges between direct neighbors from actigraphic recordings for 300 min (one min sequences), with both the directed and the undirected similarity graph, using 40 (20 + 20) neighbors. For the undirected similarity graph number of components are also given. PC = Pearson correlations, P = p-value.   \| **PC P**   \| **Directed** \| \| --- \| \| Missing edges 0.032 0.785 \| \| **Undirected** \| \| Components -0.003 0.979 \| \| Missing edges -0.130 0.264 \| \| \| --- \| --- \| --- \| --- \| --- \| --- \| \|  \| \|  \| \|  \| \|  \| \|  \| \|  \| \|  \|  \| \| --- \| \|  \| \|  \|  \| \| --- \| \|  \| \|  \| \|  \| \|  \|  \|  \| \| --- \| \|  \| \|  \| \|  \| \|  \| \| \| --- \| --- \| --- \| --- \| --- \| --- \| --- \| --- \| --- \| --- \| --- \| --- \| --- \| --- \| --- \| --- \| --- \| --- \| --- \| --- \| --- \| --- \| --- \| --- \| --- \| --- \| --- \| --- \| --- \| --- \| --- \| --- \| --- \| \|  \| \|  \| \|  \| \| \| \| --- \| --- \| --- \| --- \| --- \| --- \| --- \| --- \| --- \| --- \| --- \| --- \| --- \| --- \| --- \| --- \| --- \| --- \| --- \| --- \| --- \| --- \| --- \| --- \| --- \| --- \| --- \| --- \| --- \| --- \| --- \| --- \| --- \| --- \| --- \| --- \| --- \| --- \| --- \| --- \| --- \| --- \| --- \| --- \| \| \|  \| \| --- \| \|  \| \|  \| \|  \| \| \|  \|  \| \| --- \| \|  \| \|  \| \|  \| \|  \| \|  \| \|  \| \|  \| \|  \| |
